# Alteration of the protein quality control system in motor neuron and muscle expressing mutant proteins causing ALS and SBMA

**DOI:** 10.1186/2193-1801-4-S1-P6

**Published:** 2015-06-12

**Authors:** Maria Elena Cicardi, Valeria Crippa, Riccardo Cristofani, Paola Rusmini, Angelo Poletti

**Affiliations:** Universita Degli Studi di Milano, Italy

**Keywords:** Neurodegeneration, misfolded proteins, PQC system

Motor neuronal system and muscle tissue are two districts differently affected at onset and during the progression of diseases like amyotrophic lateral sclerosis (ALS) or spinal and bulbar muscular atrophy (SBMA). The bases of these diseases are linked to mutated proteins: fALS is commonly caused by mutations in the SOD1 or the TDP43 genes and SBMA is caused by a of CAG repeat in the AR gene. A fraction of these proteins can not reach a mature conformation and misfold. The Protein Quality Control (PQC) system is responsible for the correct protein homeostasis: the chaperones maintain proteins in their correct conformations. If they fail, mutated proteins are directed to the proteasome or the macroautophagy. When misfolded proteins are not correctly removed, they aggregate in nucleus and cytoplasm. To understand cellular behavior in presence of misfolded toxic proteins we investigate the different activation of the PQC system in the two mayor tissues involved. Initially we investigated the differences in PQC activation between NSC34 motor neuronal and C2C12 muscular cell models. Using RTq PCR, western blot and immunocytochemical analysis for p62 and LC3 expression, localization, and turnover we demonstrated that C2C12 cells have a more active autophagic system than NSC34 cells. Then, we compared the two models in presence of misfolded protein inhibiting degradative systems. With Filter Retardation Assay, we observed that these proteins tend to aggregate when PQC system is impaired. Then, we potentiated the PQC response to reduce the insoluble species. By overexpressing the small heat shock protein B8 in both systems we demonstrated that AR polyQ and SODG93A insoluble species were reduced. Also autophagy activation by trehalose caused a reduction in protein aggregation in both cell models. In conclusion misfolded protein aggregates can be reduced by modulating macroautophagy and this could represent a new therapeutical strategy for disease like SBMA and ALS.

